# A Study on the Evolution Laws of Entrainment Performances Using Different Mixer Structures of Ejectors

**DOI:** 10.3390/e26110891

**Published:** 2024-10-22

**Authors:** Hongjie Chen, Jing Ge, Zhizhou Xu

**Affiliations:** 1 School of Energy and Building Environment, Guilin University of Aerospace Technology, Guilin 541004, China; capricorn1987@126.com (H.C.); xuzhizhou_gxu@163.com (Z.X.); 2 School of Chemical Engineering, Guizhou University of Engineering Science, Bijie 551700, China

**Keywords:** ejector, cylindrical mixer, conical–cylindrical mixer, entrainment ratio, entropy generation rate

## Abstract

Being the core of the ejector refrigeration system, an ejector with a suitable mixer, conical–cylindrical or cylindrical, is key to high-energy-efficiency and low-carbon systems. To promote the scientific selection of mixers for ejectors based on the theoretical models that have been validated by experiments, the evolution laws of the entrainment ratios in the two types of ejectors are studied under various operating conditions. Furthermore, the influence mechanism of the mixer structures on the entrainment ratio of the ejector is elucidated by comparing the distribution characteristics of the entropy generation rate, pressure lift proportion, and entropy generation rate of the per-unit pressure lift in the two types of ejectors. The efficiencies of the conical-cylindrical mixer ejector and cylindrical mixer ejector exist a crossover, which makes the entrainment ratio of the conical–cylindrical mixer ejector smaller under small compression ratios but larger under large compression ratios. By changing the cylindrical mixer into a conical one, on the one hand, more pressure rise will be distributed in the diffuser, which helps to reduce the entropy increase rate in the pressurization process; on the other hand, the wall impulse effect of the conical mixer will lead to an increase in entropy generation rate of per-unit pressure lift, resulting in a growing entropy generation rate of boosting. The dominant roles are not the same with changing compression ratios, which leads to different relationships of entrainment ratio between the cylindrical and conical mixer ejectors.

## 1. Introduction

**Figure 1:**
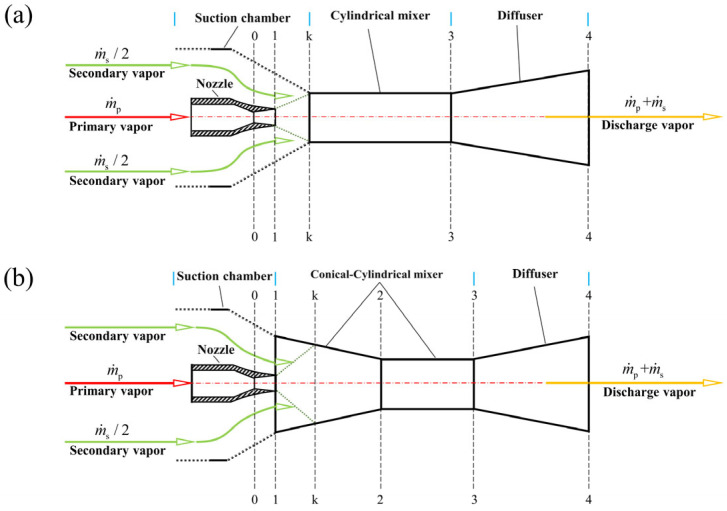
Structure of ejectors with cylindrical (**a**) and conical–cylindrical (**b**) mixers.

According to the data from China’s National Bureau of Statistics, air conditioning energy consumption accounts for about 9.6% of the total energy consumption in China [1]. In 2021, the electricity consumed by air conditioning, which was generated primarily through thermal methods, led to over 0.5 billion tons of CO_2_ emissions [2]. Therefore, under the context of “low carbon”, refrigeration systems powered by clean energy (such as solar energy) have received more and more attention [3]. In an ejector refrigeration system, the economics depend on ejector efficiency: higher efficiency means lower equipment costs [4]. Therefore, designing an efficient ejector is the key to the development of solar ejector refrigeration systems. According to the available reports, the design of the mixer structure has an important influence on the performance of the ejector [5]. To the best of the authors’ knowledge, the vapor ejectors can be divided into three types according to the structure of the mixer: the conical–cylindrical mixer ejector (CCME), the cylindrical mixer ejector (CME), and the curved profile variable area mixer ejector (e.g., the constant rate of momentum change ejector) [6]. The constant rate of momentum change ejector may perform better than the conical–cylindrical mixer ejector [7]. However, as shown in Figure 1, the vapor ejectors that are widely used in ejector refrigeration systems are the cylindrical mixer ejector and the conical–cylindrical mixer ejector [8,9]. The reason why the curved profile variable area mixer ejectors have not been widely used may be because of more complicated design calculations and higher processing costs. Thus, the cylindrical mixer and conical–cylindrical mixer ejectors have become the focus of research. The thermodynamic model development of each mixing chamber ejector is shown in Table 1 and Table 2.

Comparing Table 1 and Table 2, it is found that the cylindrical mixer ejector model is more mature than the conical–cylindrical mixer ejector model, which may be the reason why more systems currently choose cylindrical mixer ejector in engineering applications. However, the CCME may outperform the CME, prompting an increasing number of comparative studies between the two. As early as 1950 as illustrated in Table 3, Keenan et al. [23] experimentally determined that the CCME might perform better than the CME under an ejector area ratio (*Ar*) smaller than 10. In addition, Sokolov et al. [13] recommended, based on practical experience, that the CME should be used when the compression ratio (*C*) was less than 2.5 and that the CCME should be chosen when the compression ratio was larger than 2.5. To scientifically define the selection rule for CMEs and CCMEs, more and more scholars have devoted themselves to researching the entrainment ratio (*Er*) differences between CMEs and CCMEs. With a generating temperature of *T*_p_ = 88~102 °C and an evaporating temperature of *T*_s_ = 81.6 °C, the experiments by Shestopalov et al. showed that the CCME with a mixer diameter of 13.02 mm and a nozzle throat diameter of 4.21 mm had a larger entrainment ratio and lower critical back pressure (*P*_d,cri_) compared with the CME [29]. A similar conclusion was also obtained by Valle et al. [30] for the R134a ejector with a mixer diameter of 4.8 mm and a nozzle throat diameter of 4 mm under *T*_p_ = 84.39 °C and *T*_s_ = 10 °C. Considering both the same primary and secondary vapor parameters (*T*_p_ = 95 °C and *T*_s_ = 12 °C) as well as the same condensing temperature of 32 °C, a theoretical study revealed that the CCME performed better than the CME with various organic refrigerants, although the types of superiority differed for different working fluids [9]. Zhu et al. [31] showed that for the R141b ejector with a primary flow pressure of *P*_p_ = 5 bar, secondary flow pressure of *P*_s_ = 0.43 bar, and condensing pressure of *P*_d_ = 0.8 bar, the CCME with a mixer converging angle (*θ*) of 1.45° showed a conspicuous performance improvement compared with the CME.

However, previous studies comparing conical–cylindrical mixer ejectors and cylindrical mixer ejectors have primarily focused on specific operating conditions, namely, expansion ratio *E* and compression ratio *C*, and the mechanism of how the mixer structure affects the performance of the ejector across different working conditions has not been clarified [32]. In this work, based on real gas, the classical gas dynamic design models [13] for R718 ejectors with conical–cylindrical and cylindrical mixers were experimentally validated first. Then, the evolution laws of the design entrainment ratios in the CME and CCME were studied under various operating conditions using the validated models. Furthermore, for an ejector working in the ideal state, the entrainment ratio based on the isentropic hypothesis should be a certain value (the upper limit of performance) [33,34,35], unrelated to the structure of the ejector. The cylindrical mixer and the conical–cylindrical mixer play the same role of mixing and boosting, with the only difference of effectiveness. Considering that entropy generation analysis is a useful tool to evaluate the effectiveness of the working process of components [36,37], the influence mechanism of the mixer structures on the entrainment performance of the ejector is elucidated based on three aspects: analyzing the distribution characteristics of entropy generation rate (*γ*) in the two types of ejectors; examining and comparing the distribution characteristics of pressure lift proportion (PLP) and entropy generation rate of per-unit pressure lift (EGRP) in the pressure lift components of both ejector types; and comparing the total entropy generation rate between the two ejector types. This study provides a thermodynamic optimization rule for the selection of the mixer structure of the ejector, as well as for optimizing the performance of an ejector refrigeration system.

## 2. Ejector Design Model

Among the ejector models proposed in previous works, Sokolov’s work, describing the design models for the CCME and CME, respectively, is the most comprehensive one [13]. However, in addition to different mixer structures, the models rely on inconsistent assumptions for the two types of ejectors. These include the following: (1) Different assumptions are applied to the nozzle outlet pressure. (2) Additional assumptions are required for conical–cylindrical mixer ejectors about the relationship between the area of the secondary vapor choking section and the area of the mixer throat. (3) Additional assumptions are also needed for conical–cylindrical mixer ejectors regarding the relationship between the mixer inlet dimension and the mixer throat dimension. These inconsistent modeling assumptions restrict the feasibility of theoretically finding the mechanism of the performance difference between the two structures. Therefore, it is necessary to optimize the design models of the CME and the CCME first.

The following assumptions are proposed to establish the mathematical model for the ejector design in this work: (1) The velocities of the primary and secondary vapor at the entrance and the velocity of the discharge vapor are so small that they can be ignored, which can be regarded as a stagnant state. (2) The secondary vapor reaches its critical state at a specific section k within the mixer, that is, *P*_k_ = *P*_s,cri_ [13]. (3) The primary vapor and the secondary vapor at the outlet of the nozzle have the same pressure at *P*_1_ [38]. (4) The flow of the vapors in the ejector can be regarded as adiabatic. The mathematical models of the CME and CCME are described below.

### 2.1. Model of Ejector with a Cylindrical Mixer

**Figure 2:**
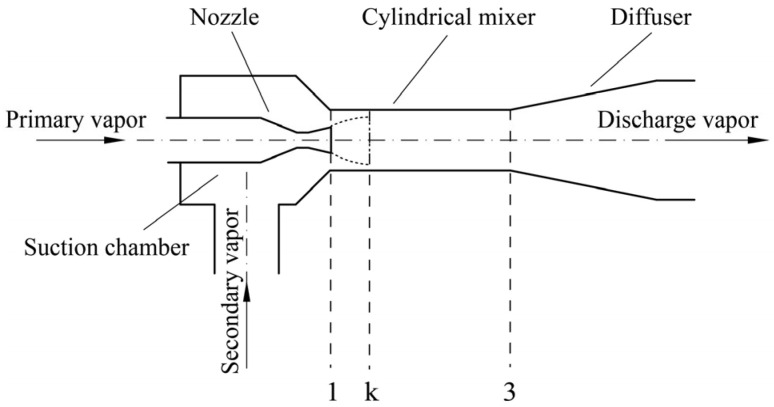
Structure of the cylindrical mixer ejector.

For the CME, as shown in Figure 2, considering the momentum loss due to the frictional resistance of the mixer, the momentum conservation equation of the control volume between sections 1 and 3 can be described as:

(1) $$\varphi \left({\dot{m}}_{p}w_{p,1}+{\dot{m}}_{s}w_{s,1}\right)-\left({\dot{m}}_{p}+{\dot{m}}_{s}\right)w_{d,3}=P_{d,3}A_{d,3}-P_{1}A_{p,1}-P_{1}A_{s,1}$$

where the momentum efficiency *η* is 0.975 [13]. The non-isentropic expansion and compression processes should be corrected by the velocity coefficient; therefore, the velocities of the primary vapor and the secondary vapor at the nozzle exit and the velocity of the vapor at the mixer outlet are respectively expressed as:

(2) $$w_{p,1}=\eta_{\mathrm{noz}}w_{p,\mathrm{cri}}\lambda_{p,1}$$

(3) $$w_{s,1}=\eta_{\mathrm{suc}}w_{s,\mathrm{cri}}\lambda_{s,1}$$

(4) $$w_{d,3}=\frac{w_{d,\mathrm{cri}}}{\eta_{\mathrm{dif}}}\lambda_{d,3}$$

The isentropic efficiencies of the nozzle, diffuser, and suction chamber (*η*_noz_, *η*_dif_, and *η*_suc_) can take the values of 0.975, 0.9, and 0.925, respectively [13]. The areas occupied by the primary vapor and the secondary vapor in section 1 can be calculated by:

(5) $$A_{p,1}=\frac{{\dot{m}}_{p}w_{p,\mathrm{cri}}}{k_{p}\Pi_{p,\mathrm{cri}}P_{p}q_{p,1}}$$

(6) $$A_{s,1}=\frac{{\dot{m}}_{s}w_{s,\mathrm{cri}}}{k_{s}\Pi_{s,\mathrm{cri}}P_{s}q_{s,1}}$$

The area of section 1 is occupied by the primary vapor and the secondary vapor:

(7) $$A_{1}=A_{p,1}+A_{s,1}$$

The area of the mixer outlet can be expressed by the equation of continuity as:

(8) $$A_{d,3}=\frac{\left({\dot{m}}_{p}+{\dot{m}}_{s}\right)w_{d,\mathrm{cri}}}{k_{d}\Pi_{d,\mathrm{cri}}P_{d}q_{d,3}}$$

For a cylindrical mixer, the inlet and outlet should meet the relationship of:

(9) $$A_{1}=A_{d,3}$$

The entrainment ratio can be expressed as:

(10) $$Er=\frac{{\dot{m}}_{s}}{{\dot{m}}_{p}}$$

The ejector should operate at the intersection of the single-choking and double-choking states under the given design conditions [39]. According to the assumption, the double-choking state means that at a certain section, such as section k, the velocity of the secondary vapor reaches its critical state. According to the continuity equation in section k, the entrainment ratio in the double-choking state, *Er*_cho_, can be determined by:

(11) $$Er_{\mathrm{cho}}=\frac{\frac{w_{d,\mathrm{cri}}}{w_{s,\mathrm{cri}}}\frac{k_{s}}{k_{d}}\frac{\Pi_{s,\mathrm{cri}}}{\Pi_{d,\mathrm{cri}}}\frac{P_{s}}{P_{d}}\frac{1}{q_{d,3}}-\frac{w_{p,\mathrm{cri}}}{w_{s,\mathrm{cri}}}\frac{k_{s}}{k_{p}}\frac{\Pi_{s,\mathrm{cri}}}{\Pi_{p,\mathrm{cri}}}\frac{P_{s}}{P_{p}}\frac{1}{q_{p,\;k}}}{1-\frac{w_{d,\mathrm{cri}}}{w_{s,\mathrm{cri}}}\frac{k_{s}}{k_{d}}\frac{\Pi_{s,\mathrm{cri}}}{\Pi_{d,\mathrm{cri}}}\frac{P_{s}}{P_{d}}\frac{1}{q_{d,3}}}$$

Meanwhile, because the primary vapor also chokes at the throat of the nozzle, there is:

(12) $$A_{p,0}=\frac{{\dot{m}}_{p}w_{p,\mathrm{cri}}}{k_{p}\Pi_{p,\mathrm{cri}}P_{p}}$$

After the entrainment ratio is determined, the energy conservation equation of the ejector can be expressed by:

(13) $$h_{d}=\frac{h_{p}+Er\cdot h_{s}}{1+Er}$$

### 2.2. Model of Ejector with a Conical–Cylindrical Mixer

**Figure 3:**
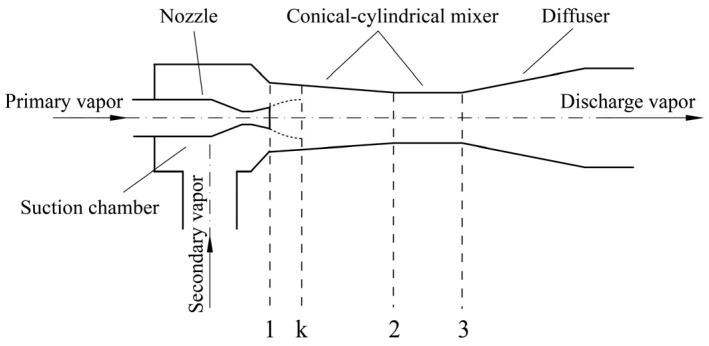
A structure of the conical–cylindrical mixer ejector.

The premise of the mathematical model for the CCME is the same as that for the CME. As shown in Figure 3, the equation of momentum conservation is presented for the mixer:

(14) $$\varphi \left({\dot{m}}_{p}w_{p,1}+{\dot{m}}_{s}w_{s,1}\right)-\left({\dot{m}}_{p}+{\dot{m}}_{s}\right)w_{d,3}=P_{d,3}A_{d,3}+\int_{A_{1}}^{A_{d,3}}PdA-P_{1}A_{p,1}-P_{1}A_{s,1}$$

The integral term in the momentum conservation equation can be expressed as:

(15) $$\int_{A_{1}}^{A_{d,3}}PdA=\frac{A_{d,3}\left(\beta -1\right)}{2}\left[P_{1}+P_{d,3}{\left(\frac{P_{s}\Pi_{s,1}}{P_{d}\Pi_{d,3}}\right)}^{\alpha }\right]$$

where *α* is the ratio of the pressure lift in the conical part to the total pressure lift in the conical–cylindrical mixer, with the value of 0.5 in the model. For a conical–cylindrical mixer, the relationship between the inlet and outlet flow area is:

(16) $$A_{1}=\beta A_{d,3}$$

The entrainment ratio of the CCME is also restricted by the double-choking state. Considering that the secondary vapor choking section k is located in the conical mixer, the relationship between section k and the mixer throat should be as follows:

(17) $$A_{k}=\mu A_{d,3}$$

So, the double-choking entrainment ratio of the CCME can be determined by:

(18) $$Er_{\mathrm{cho}}=\frac{\mu \frac{w_{d,\mathrm{cri}}}{w_{s,\mathrm{cri}}}\frac{k_{s}}{k_{d}}\frac{\Pi_{s,\mathrm{cri}}}{\Pi_{d,\mathrm{cri}}}\frac{P_{s}}{P_{d}}\frac{1}{q_{d,3}}-\frac{w_{p,\mathrm{cri}}}{w_{s,\mathrm{cri}}}\frac{k_{s}}{k_{p}}\frac{\Pi_{s,\mathrm{cri}}}{\Pi_{p,\mathrm{cri}}}\frac{P_{s}}{P_{p}}\frac{1}{q_{p,\;k}}}{1-\mu \frac{w_{d,\mathrm{cri}}}{w_{s,\mathrm{cri}}}\frac{k_{s}}{k_{d}}\frac{\Pi_{s,\mathrm{cri}}}{\Pi_{d,\mathrm{cri}}}\frac{P_{s}}{P_{d}}\frac{1}{q_{d,3}}}$$

**Figure 4:**
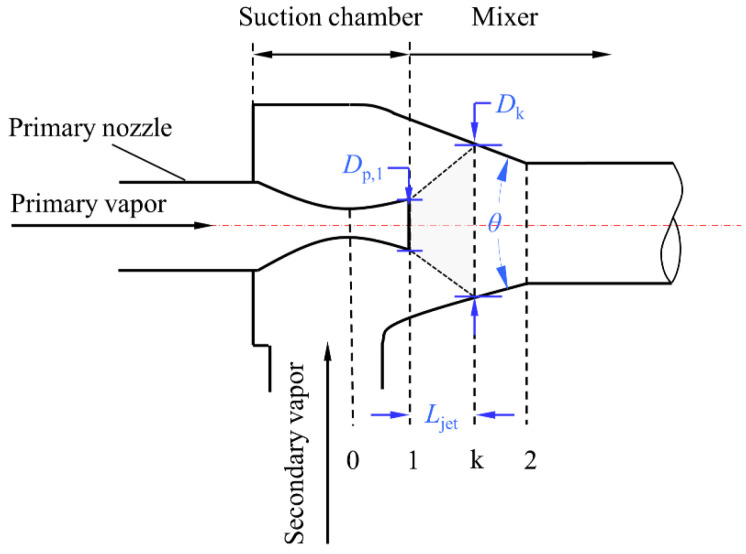
The flow in the inlet region of the conical–cylindrical mixer.

The calculation of the entrainment ratio for the CCME involves the values of *μ* and *β*. To determine *μ* and *β*, we need to calculate the inlet cross-sectional area of the mixer (*A*_1_) and the area of the secondary vapor choking section (*A*_k_). As shown in Figure 4, both *A*_1_ and *A*_k_ can be calculated based on specific cases.

In the case of *Er* ≥ 0.5, the length of the jet flow (*L*_jet_) and the diameter of the secondary vapor choking section (*D*_k_) can be calculated by [13]:

(19) $$L_{\mathrm{jet}}=1.05+2.84Er$$

(20) $$D_{k}=1.55D_{p,1}\left(1+Er\right)$$

In the case of *Er* ≤ 0.5, the *L*_c_ and the *D*_k_ are respectively expressed as [9]:

(21) $$L_{\mathrm{jet}}=\left(\sqrt{3.237+29.64Er}-1.81\right)D_{p,1}$$

(22) $$D_{k}=D_{p,1}\sqrt{0.96+8.79Er}$$

According to the characteristic of the conical mixer, the inlet diameter of the mixer (*D*_1_) and the diameter of the secondary vapor choking section meet the relation of:

(23) $$D_{1}=D_{k}+2L_{\mathrm{jet}}\tan\frac{\theta }{2}$$

where *θ* is the convergence angle, taking the recommended value of 6° [40].

### 2.3. Ejector Design Steps

#### 2.3.1. Design Calculation of the CME

**Figure 5:**
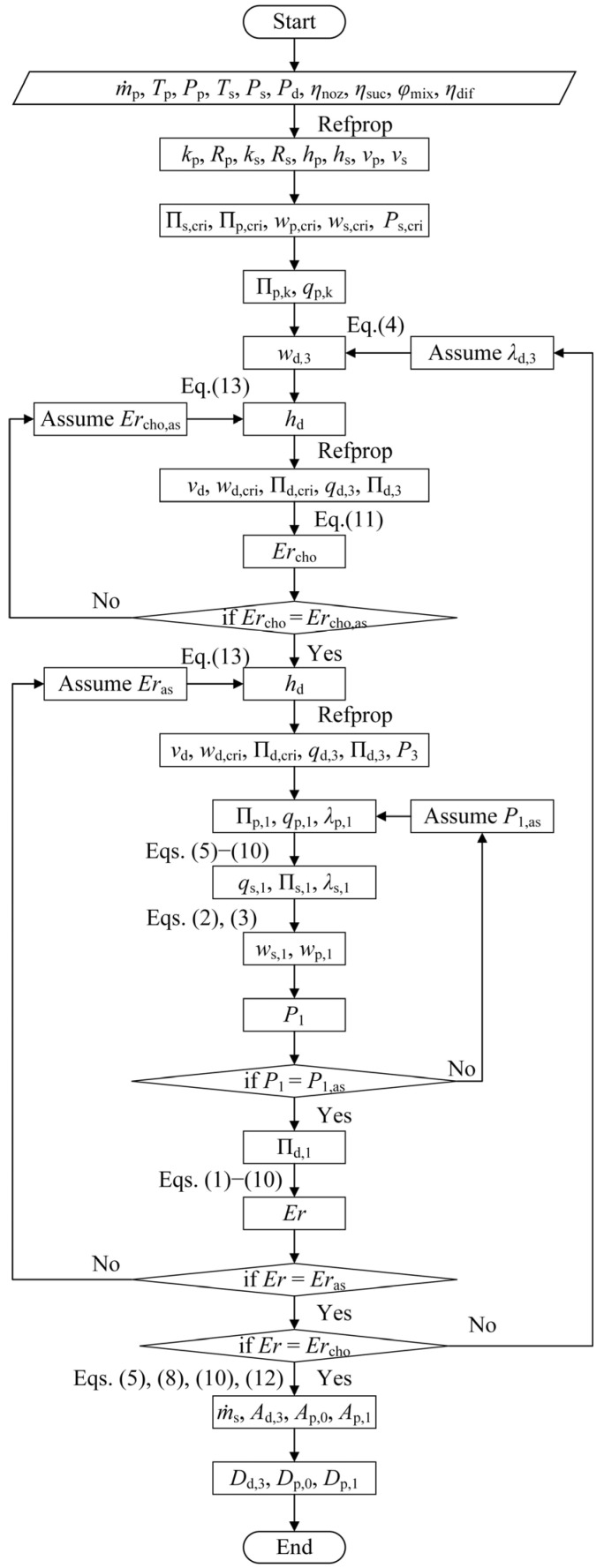
A calculation flow chart of the ejector with the cylindrical mixer.

As presented in Figure 5, for a CME, the design steps are as follows: (1) Determine the specific volume, specific enthalpy, adiabatic index, and gas constant using the temperature and pressure of the primary and secondary vapor. (2) Calculate *w*_p,cri_ and *Π*_p,cri_ for the primary vapor and *w*_s,cri_, *Π*_s,cri_ and P_s,cri_ for the secondary vapor using gas dynamic functions. (3) Given that *P*_k_ = *P*_s,cri_ on the secondary vapor choking section, calculate *Π*_p,k_ and *q*_p,k_. (4) Assume a value for *λ*_d,3_. (5) Calculate w_d,3_ using Equation (4). (6) Assume a value for *Er*_cho,as_. (7) Calculate h_d_ using Equation (13). (8) Use the Refprop software (V9.1) to obtain vd based on *P*_d_ and *h*_d_. Then, use gas dynamic functions to calculate *w*_d,cri_ and *Π*_d,cri_ for the vapor, as well as *q*_d,3_ and *Π*_d,3_ for the mixing vapor. (9) Calculate *Er*_cho_ using Equation (11). If *Er*_cho_ is not equal to the hypothetical value *u*_cho,as_, return to Step 6. (10) Assume a value for *u*_as_. (11) Calculate *h*_d_ again using Equation (13). (12) Find *v*_d_ again based on *P*_d_ and *h*_d_. Use gas dynamic functions to calculate *w*_d,cri_ and *Π*_d,cri_, as well as *q*_d,3_ and *Π*_d,3_. Then, determine *P*_3_. (13) Assume a value for the pressure *P*_1,as_. (14) Calculate *Π*_q,1_, *q*_p,1_, and *λ*_p,1_ using gas dynamics functions. (15) Calculate *Π*_s,1_, *q*_s,1_, and *λ*_s,1_ using Equations (5)–(10). (16) Determine *w*_p,1_ and *w*_s,1_ using Equations (2) and (3). Simultaneously, calculate *P*_1_. If *P*_1_ is not equal to *P*_1,as_, reassume *P*_1_ and return to Step 13. (17) Calculate *Π*_d,1_ using the gas dynamic method. (18) Calculate the entrainment ratio *Er* using Equations (1)–(10). If *Er* is not equal to *Er*_as_, reassume *Er*_as_ and return to Step 10. (19) Compare *Er* with *Er*_cho_. If they are not equal, reassume *λ*_d,3_ and repeat Steps 5 to 18. (20) Calculate *A*_p,1_, *A*_d,3_, *ṁ*_s_, and *A*_p,0_ using Equations (5), (8), (10) and (12). (21) Finally, use the relationship between the area and the diameter to obtain *D*_d,3_, *D*_p,0_, and *D*_p,1_.

#### 2.3.2. Design Calculation of the CCME

**Figure 6:**
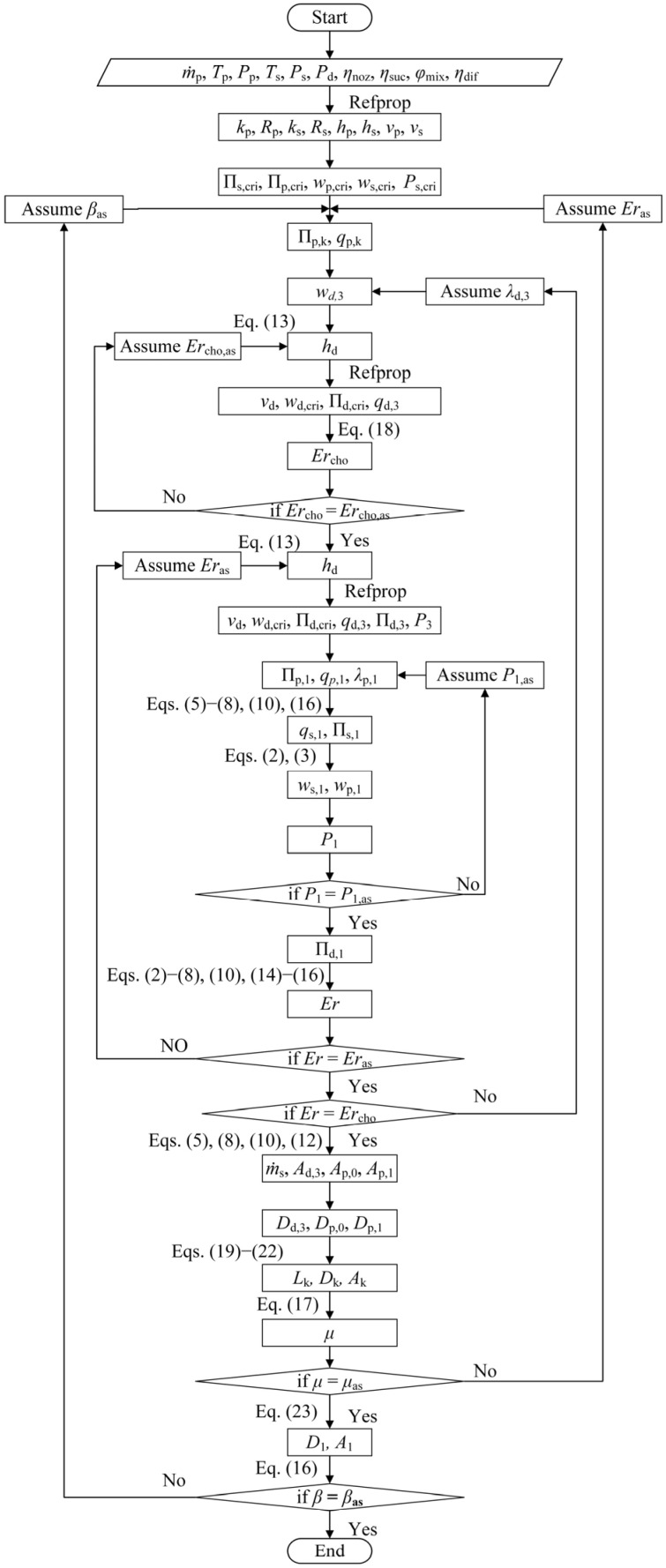
A calculation flow chart of the ejector with the conical–cylindrical mixer.

The design steps of the CCME are outlined in Figure 6: (1) Determine the specific volume, specific enthalpy, adiabatic index, and gas constant of the primary and secondary vapor from their respective temperatures and pressures. (2) Use gas dynamics functions to calculate *w*_p,cri_ and *Π*_p,cri_ for the primary vapor. Similarly, determine *w*_s,cri_, *Π*_s,cri_, and *P*_s,cri_ for the secondary vapor. (3) Given that *P*_k_ = *P*_s,cri_ on the secondary vapor choking section, use gas dynamics functions to obtain *Π*_p,k_ and *q*_p,k_. (4) Assume a value for *β*_as_. (5) Assume a value for *μ*_as_. (6) Assume a value for *λ*_d,3_. (7) Calculate *w*_d,3_ using Equation (4). (8) Assume a value for *Er*_cho,as_. (9) Calculate *h*_d_ using Equation (13). (10) Determine *v*_d_ based on *P*_d_ and *h*_d_. Then, use gas dynamic functions to calculate *w*_d,cri_ and *Π*_d,cri_ for the discharge vapor, as well as *q*_d,3_ and *Π*_d,3_. (11) Calculate *Er*_cho_ using Equation (11). If *Er*_cho_ is not equal to the hypothetical value of *Er*_cho,as_, assume a new *Er*_cho,as_ and return to Step 8. (12) Assume a value for *Er*_as_. (13) Calculate *h*_d_ again using Equation (13). (14) Determine *v*_d_ again based on *P*_d_ and *h*_d_. Use gas dynamic functions to calculate *w*_d,cri_ and *Π*_d,cri_ for the discharge vapor, and *q*_d,3_ and *Π*_d,3_ for the mixing vapor at the mixer outlet. (15) Assume a value for the pressure *P*_1,as_. (16) Calculate *Π*_p,1_, *q*_p,1_, and *λ*_p,1_ using gas dynamics functions. (17) Use Equations (5)–(8), (10) and (16) to calculate *Π*_s,1_ and *q*_s,1_. (18) Use Equations (2) and (3) to calculate *w*_p,1_ and *w*_s,1_. Simultaneously, obtain *P*_1_. If *P*_1_ is not equal to *P*_1,as_, reassume *P*_1,as_ and return to Step 15. (19) Use gas dynamic functions to calculate *Π*_d,1_. (20) Use Equations (2)–(8), (10) and (14)–(16) to calculate *Er*. If *Er* is not equal to *Er*_as_, reassume *Er*_as_ and return to Step 12. (21) Compare *Er* with *Er*_as_. If they are not equal, reassume *λ*_d,3_ and repeat Steps 6 to 20. (22) Calculate *ṁ*_s_, *A*_d,3_, *A*_p,0_, and *A*_p,1_ using Equations (5), (8), (10) and (12). Then, use the relationship between the area and the diameter to obtain *D*_d,3_, *D*_p,0_, and *D*_p,1_. (23) Based on the value of *Er*, select the appropriate Equations (19)–(22) to calculate *L*_jet_ and *D*_k_. Then, calculate the area *A*_k_. (24) Use Equation (17) to obtain *μ*. Compare *μ* with *μ*_as_. If they are not equal, assume a new *μ*_as_ and return to Step 5. (25) Use Equation (23) to calculate *D*_1_ and then calculate the area *A*_1_. (26) Use Equation (16) to calculate *β*. Compare *β* with the hypothetical value *β*_as_. If they are not equal, the calculation ends. Otherwise, reassume *β*_as_ and return to Step 4.

Compared with the referenced model by Sokolov and Zinger [13], the improved model can theoretically formulate the parameters *μ* and *β*, which is crucial for determining the entrainment ratio and back pressure under design conditions. For both the CME and CCME, the key diameters can be determined using the aforementioned steps, and the dimensions of other structures can be ascertained using the information provided in the literature [40].

## 3. Experimental Validation

### 3.1. Experimental Materials

**Figure 7:**
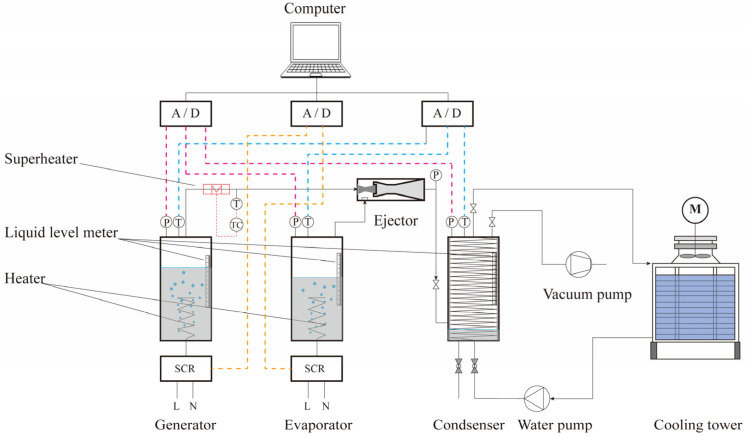
A schematic diagram of ejector performance experiment.

To validate the revised CME and CCME design models, the ejector performance test system depicted in Figure 7 is proposed. In this system, water in the evaporator absorbs heat and evaporates at low pressure, entering the ejector as the secondary vapor. Meanwhile, water in the generator absorbs heat and evaporates at high pressure, entering the ejector as the primary vapor. After passing through the ejector, the combined vapor stream flows out and enters the condenser where it releases heat and becomes a condensate. The system is open, meaning that the vapors from the generator and evaporator enter the condenser and ultimately remain there. Under stable operating conditions, the entrainment ratio of the ejector can be determined by measuring the reduction in liquid level in both the generator and the evaporator over a specified period of time.

**Figure 8:**
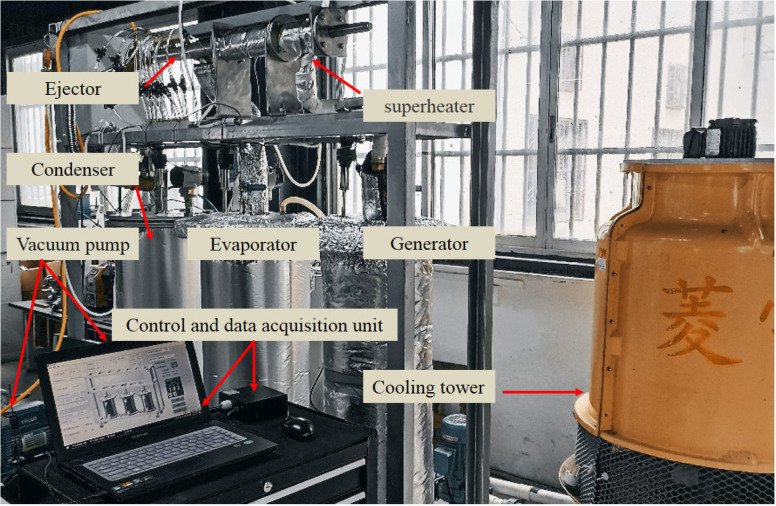
A device for the ejector performance test.

As shown in Figure 8, the main devices—the generator, evaporator, condenser, and cooling tower—are produced by Guilin Boao company. The heat required by the generator is supplied by a variable-power electric heater; the power of the heater is adjusted through thyristor voltage regulation to achieve the target generating pressure. The pressure of the evaporator is also stabilized in a similar manner. The condenser is a shell-and-tube heat exchanger that is cooled by water from the cooling tower. The condensation pressure is adjusted by regulating the flow of cooling water. Water, which is used as a refrigerant in air conditioning systems, has zero ozone depletion potential and zero global warming potential; furthermore, it is inexpensive and easily accessible [41]. Therefore, water is the preferred refrigerant option in this study.

In the measuring device, the liquid level is tested using a glass-tube liquid-level gauge, which has a scale length of 500 mm and a minimum scale division of 1 mm. The sensors used for temperature testing are PT100 sensors from Anhui Kexun, with measuring ranges of 0~150 °C and 0~100 °C, and accuracies of 0.375 °C and 0.25 °C, respectively. For pressure measurement, single-crystal silicon pressure sensors from Hangzhou Meacon with the parameters listed in Table 4 are adopted.

The stability of the experimental system was ensured using a reliable automatic control system. To further illustrate the reliability of the experimental results, a comprehensive uncertainty [42] was introduced to evaluate the key experimental measurement parameters (*Er* and *C*). The uncertainty analysis results are listed in Table 5. Clearly, the largest uncertainty of 2.0% occurred in the condenser, which implies that the system stability and instrument reliability are acceptable.

### 3.2. The Ejectors Used in the Experiment

**Figure 9:**
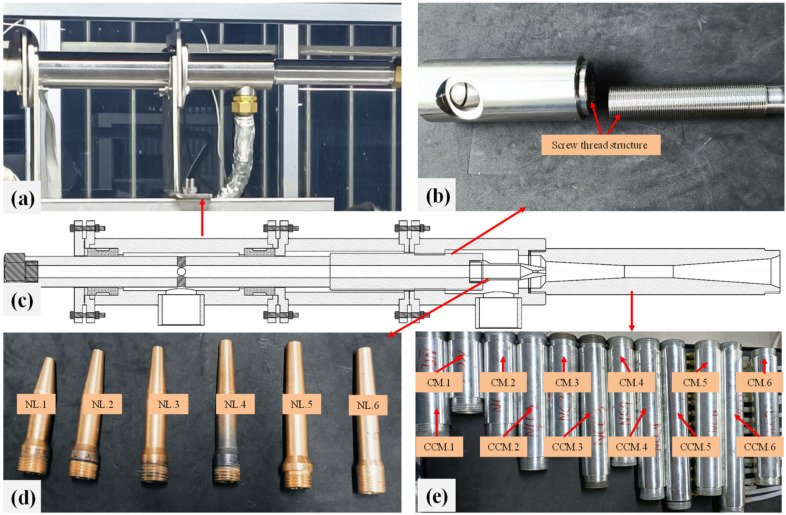
The detachable ejector for experiments: (**a**) Ejector body; (**b**) Screw thread structure; (**c**) Ejector structure design drawing; (**d**) Nozzles; (**e**) Mixing–diffusing chambers.

**Figure 10:**
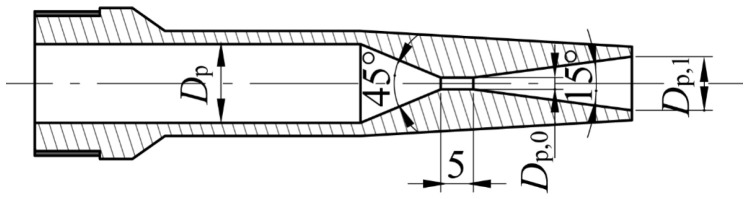
A structure of the nozzle.

**Figure 11:**
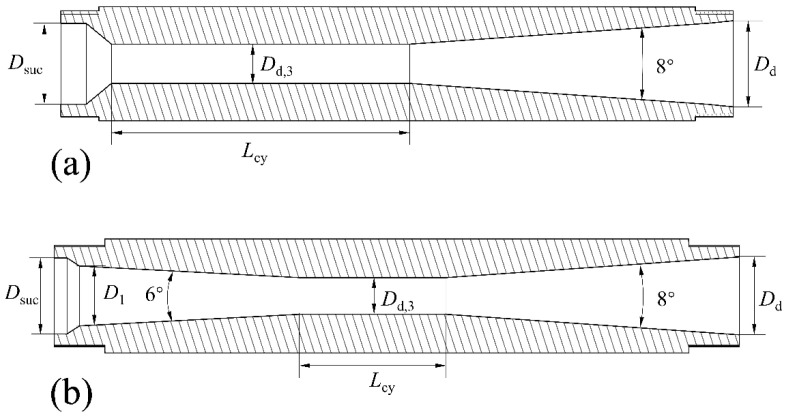
A structure of the mixing–diffusing chamber with cylindrical (**a**) and conical–cylindrical (**b**) mixer.

The ejector used in the experiment features a detachable structure (see Figure 9). The main body of the ejector can be reused, while only the nozzle and the mixing–diffusing chamber need to be replaced for each test. The structures of the nozzle, cylindrical mixer, and conical–cylindrical mixer are illustrated in Figure 10 and Figure 11. For different working conditions, six groups of CMEs and CCMEs have been designed, with detailed dimensions listed in Table 6, Table 7 and Table 8. For the dimensions listed, the allowable tolerance is ±0.05 mm for dimensions less than 6 mm, ±0.1 mm for dimensions between 6 mm and 30 mm, and ±0.15 mm for dimensions greater than 30 mm [43].

### 3.3. Analysis of the Experimental Result

The experimental results are shown in Table 9 and Table 10. When comparing the experimental values with the predicted data, it can be found that the relative deviations of the entrainment ratio and critical back pressure for the CME are within ±12.2% and ±3.82%, respectively. The average absolute values of these relative deviations are 5.11% and 1.93%, respectively. Similarly, for the CCME, the relative deviations of the entrainment ratio and critical back pressure are within ±3.86% and ±2.53%, with average absolute values of 2.14% and 1.92%, respectively. The design models for both the CME and CCME are considered reliable.

Notably, when comparing the experiments of the CME and CCME, the results reveal that the entrainment ratios of CCM. 1, CCM. 2, CCM. 3, CCM. 4, CCM. 5, and CCM. 6 increased by 21.94%, 34.30%, 40.96%, 42.40%, 59.68%, and 84.26%, respectively, compared with those of CM. 1, CM. 2, CM. 3, CM. 4, CM. 5, and CM. 6. However, the deviations of the critical back pressures are −9.54%, −3.91%, −4.43%, −8.57%, −9.24%, and −13.3%, respectively.

Clearly, when the expansion ratio is greater than 57.07, the larger the expansion ratio, the more obvious the advantage of the entrainment ratio (*Er*) in the CCME compared with the CME. Meanwhile, the advantage of the critical back pressure (*P*_d,cri_) in the CME is more pronounced compared with the CCME. However, when the expansion ratio E is less than 57.07, as the expansion ratio decreases, the relative advantages of both *Er* and *P*_d,cri_ in the CCME compared with the CME continue to diminish. This interesting experimental phenomenon may suggest that both Er and *P*_d,cri_ of the CME are potentially better than those of the CCME at smaller expansion ratios.

## 4. Analysis and Discussion

### 4.1. Evolution Laws of Entrainment Performances for the CME and CCME

**Figure 12:**
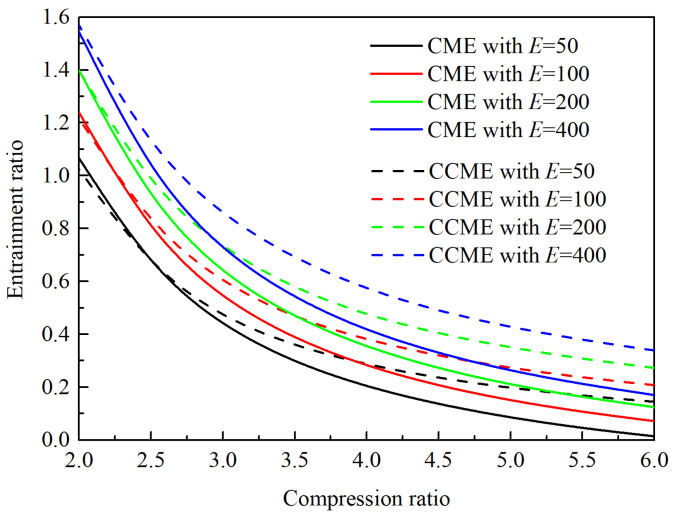
Performance comparison of the CME and CCME.

The entrainment performance characteristics of the CME and CCME are analyzed under typical ejector cooling conditions. As shown in Figure 12, it can be observed that in both the CME and the CCME, the entrainment ratio increases with an increasing expansion ratio and decreases with a growing compression ratio. It is worth noting that for each expansion ratio, there exists a specific compression ratio at which the CME and the CCME may achieve the same entrainment ratio. When the compression ratio is lower than this specific value, the CME requires a higher compression ratio to match the entrainment ratio of the CCME. Conversely, when the compression ratio is higher than this value, the CCME performs better (although under an expansion ratio of 400, this phenomenon is not directly observable due to the limited range of analyzed compression ratios). In other words, the subsequent analysis results are primarily applicable to situations where the expansion ratio is small (*E* < 400), a condition that is often met in the application of ejectors used for ejector refrigeration systems. Additionally, theoretical results clearly indicate that as the compression ratio increases, the advantage of CCME’s high efficiency becomes more evident. Furthermore, the higher the expansion ratio, the lower the compression ratio at the crossover point, which further highlights the advantage of the CCME.

### 4.2. The Mechanism of the Mixer Effecting the Entrainment Ratio

To identify the cause of the entrainment ratio crossover between the CME and CCME in the design state, the entropy generation rate (*γ*) [44] is employed as the thermodynamic basis for evaluating the entrainment performance. Subsequently, the influence mechanism of the mixer structures on the entrainment performance of the ejector is elucidated from three aspects.

#### 4.2.1. Distribution Characteristics of the Entropy Generation Rates in the CME and CCME

From a thermodynamic perspective, the selection of different mixer structures can result in variations in the degree of entropy generation, which subsequently affects the entrainment performance of an ejector. Ideally, the flow within the ejector should be isentropic. When energy conservation is taken into account, the following relationship can be established [45]:

(24) $$\left(1+Er_{\mathrm{id}}\right)\cdot s_{d}=s_{p}+Er_{\mathrm{id}}\cdot s_{s}$$

(25) $$\left(1+Er_{\mathrm{id}}\right)\cdot h_{d}=h_{p}+Er_{\mathrm{id}}\cdot h_{s}$$

In addition, the variables *s*_d_, *h*_d_, and *P*_d_ exhibit a specific thermodynamic state relationship, which can be described as follows:

(26) $$h_{d}=f\left(P_{d},s_{d}\right)$$

Since the values of *h*_p_, *s*_p_, *h*_s_, *s*_s_, and *P*_d_ are all known under design working conditions, the ideal entrainment ratio (*Er*_id_) can be determined based on Equations (24)–(26). It can also be observed that, in ideal conditions, the use of a CM or a CCM does not affect the entrainment ratio of the ejector. However, in practice, the flow within the ejector is not isentropic, and the presence of entropy generation is the direct factor causing the performance difference between the CME and the CCME. To analyze the reasons behind this performance difference, it is feasible to compare the entropy generation rates that occur in the components of the two types of ejectors. Specifically, the entropy generation rates in the nozzle, suction chamber, mixer, and diffuser chamber can be expressed as follows:

(27) $$\gamma_{\mathrm{noz}}=\frac{{\dot{m}}_{p}\left(s_{p,1}-s_{p}\right)}{{\dot{m}}_{p}s_{p}+{\dot{m}}_{s}s_{s}}$$

(28) $$\gamma_{\mathrm{suc}}=\frac{{\dot{m}}_{s}\left(s_{s,1}-s_{s}\right)}{{\dot{m}}_{p}s_{p}+{\dot{m}}_{s}s_{s}}$$

(29) $$\gamma_{\mathrm{mix}}=\frac{\left({\dot{m}}_{p}+{\dot{m}}_{s}\right)s_{d,3}-\left({\dot{m}}_{p}s_{p,1}+{\dot{m}}_{s}s_{s,1}\right)}{{\dot{m}}_{p}s_{p}+{\dot{m}}_{s}s_{s}}$$

(30) $$\gamma_{\mathrm{dif}}=\frac{\left({\dot{m}}_{p}+{\dot{m}}_{s}\right)s_{d}-\left({\dot{m}}_{p}+{\dot{m}}_{s}\right)s_{d,3}}{{\dot{m}}_{p}s_{p}+{\dot{m}}_{s}s_{s}}$$

For the specific entropy of the vapor at the inlet and outlet of the ejector, as well as the specific entropy of the primary vapor and secondary vapor at the inlet section of the mixer and the specific entropy of the vapor at the outlet of the mixer, their values can be obtained using Refprop (V9.1) [46]. Specifically, *s*_p_ is determined by *T*_p_ and *P*_p_; *s*_s_ is determined by *T*_s_ and *p*_s_; *s*_d_ can be obtained through *P*_d_ and *h*_d_; *s*_p,1_ depends on *h*_p,1_ and *P*_1_; *s*_s,1_ can be calculated using the known *h*_s,1_ and *P*_1_; and *s*_d,3_ can be determined by giving *h*_d,3_ and *P*_3_. During the calculation process, the required values of *h*_p,1_, *h*_s,1_, and *h*_d,3_ can be obtained based on energy conservation relationships, which are as follows:

(31) $$h_{p,1}=h_{p}-\frac{1}{2}w_{p,1}^{2}$$

(32) $$h_{s,1}=h_{s}-\frac{1}{2}w_{s,1}^{2}$$

(33) $$h_{d,3}=h_{d}-\frac{1}{2}w_{d,3}^{2}$$

For the ejector equipped with a cylindrical mixer, the additional parameters necessary to compute the entropy generation rate in the nozzle, suction chamber, mixer, and diffuser are derived from the flow chart presented in Figure 5. Similarly, for the ejector with a conical–cylindrical mixer, these parameters are sourced from Figure 6.

**Figure 13:**
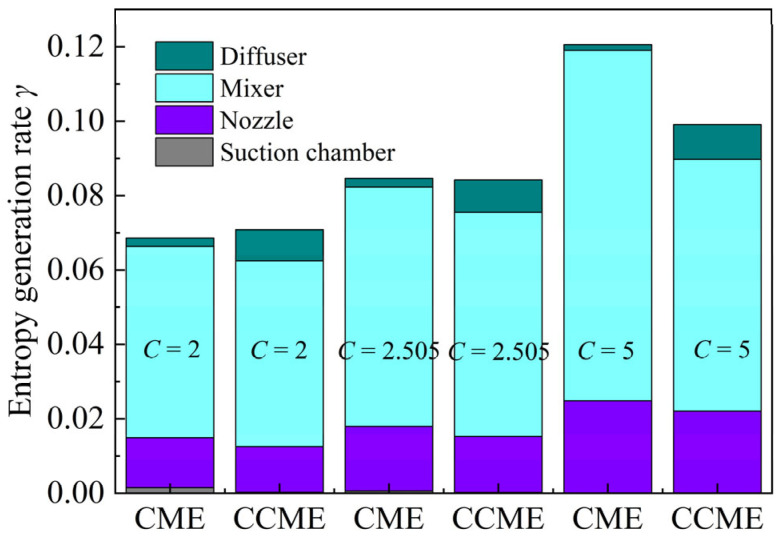
Entropy generation ratios in each part of the CME and CCME with *E* = 50.

The ejectors with expansion ratios of 50 and compression ratios of 2, 2.505, and 5 were analyzed using the above method, as shown in Figure 13. From the analysis depicted in the figure, it is evident that irrespective of whether it is a CCME or a CME, the entropy generation rate during the operational process follows the sequence of the mixer, nozzle, diffuser chamber, and suction chamber, in descending order. Notably, for CMEs, the entropy increase within the mixing chamber can surpass 70%, aligning with the findings analyzed in references [47,48].

By comparison, it was found that, after changing the mixer from cylindrical to conical–cylindrical, the entropy generation rates in the suction chamber, nozzle, mixer, and diffuser of these three groups of ejectors changed. For each group of ejectors, changing the cylindrical mixer to a conical one slightly reduced the entropy generation rate in the nozzle and suction chamber while significantly reducing the entropy generation rate in the mixer. At the same time, it obviously increased the entropy generation rate in the diffuser. Although variations in the inlet diameter of the mixer can lead to differing entropy generation rates in the nozzle and suction chamber, the primary factor influencing the overall entropy generation rate is the substantial change in entropy generation rates observed in the mixer and diffuser following alterations to the mixer structure, as illustrated in Figure 13. This further implies that, from a thermodynamic perspective, the mixer exerts a significant influence on the entrainment performance.

#### 4.2.2. Distribution Characteristics of PLP and EGRP in the Mixers and Diffusers of the CME and CCME

Considering that both the mixer and the diffuser in the two types of ejectors serve the function of pressure lift, the distribution characteristics of the pressure lift proportion (PLP) and entropy generation rate (EGRP) in the pressure lift components (mixer and diffuser) may change after altering the structure of the mixer.

Note that Equation (34) defines the PLP, and Equation (35) defines the EGRP, both in the mixer and diffuser.

(34) $${\mathrm{PLP}}_{X}=\frac{\Delta P_{X}}{P_{d}-P_{1}}$$

(35) $${\mathrm{EGRP}}_{X}=\frac{\gamma_{X}}{\Delta P_{X}}$$

where X ∈ {mix, dif} represents the different parts (mixer or diffuser) in an ejector.

**Figure 14:**
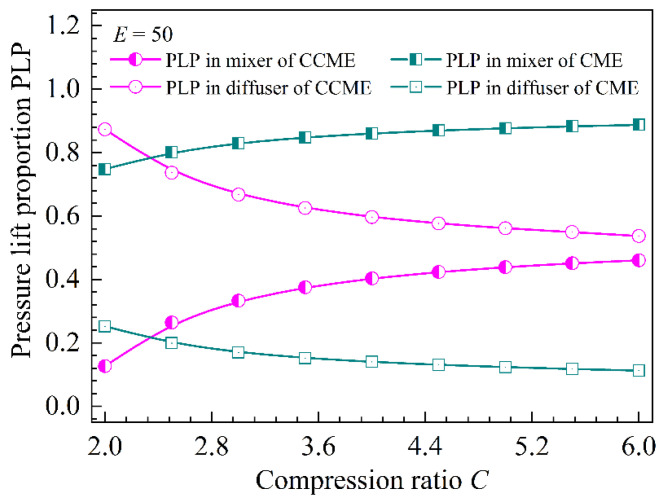
The pressure lift proportion as a function of the compression ratio in the mixer and diffuser.

For the CME and CCME with *E* = 50, Figure 14 shows the pressure lift proportions (PLP) distributed in the mixer and diffuser at different compression ratios. As the compression ratio increases, the PLP in the mixer of both the CME and CCME increases slowly, while the PLP in the diffuser gradually decreases. In addition, the PLP in the mixer of the CME is larger than that in the mixer of the CCME, while in the diffuser, the PLP of the CME is smaller than that of the CCME.

**Figure 15:**
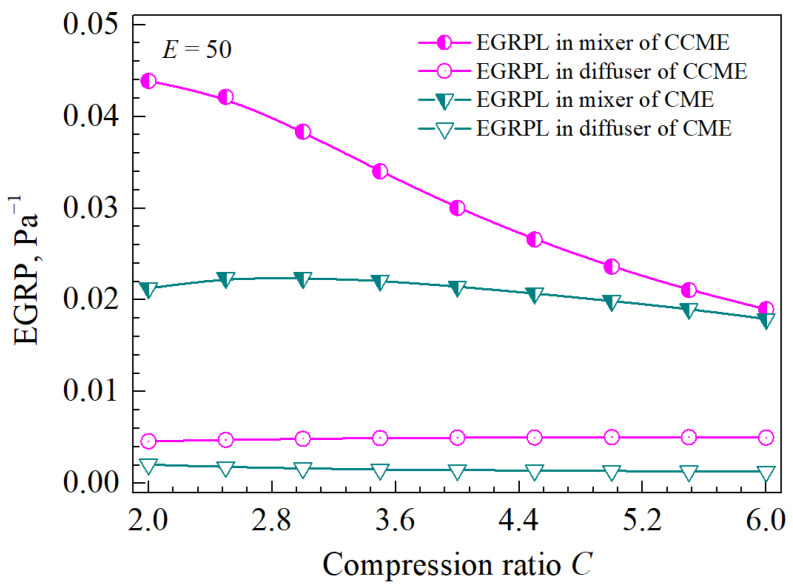
The EGRPs in the mixer and diffuser of the two types of ejectors under different compression ratios.

As the compression ratio increases (see Figure 15), the values of EGRP in the diffuser of the CCME and in the mixer and diffuser of the CME remain almost constant; however, in the mixer of the CCME, the EGRP decreases rapidly. When the compression ratio is small, the EGRP in the mixer of the CCME is much higher than that of the CME. As the compression ratio increases, the EGRP in the mixer of the CCME decreases and approaches that of the CME.

After replacing the cylindrical mixer with a conical–cylindrical one, when facing the same pressure lift, the mixer would produce a higher entropy generation rate, while the pressure previously located in the CME’s mixer (before replacement) now transfers to the diffuser, thereby reducing the entropy generation rate from the other side. As a result, this leads to an indeterminate efficiency relationship between the pressurization processes of the CME and CCME.

#### 4.2.3. Effect of Compression Ratio on the Total Entropy Generation Rate in the CME and CCME

**Figure 16:**
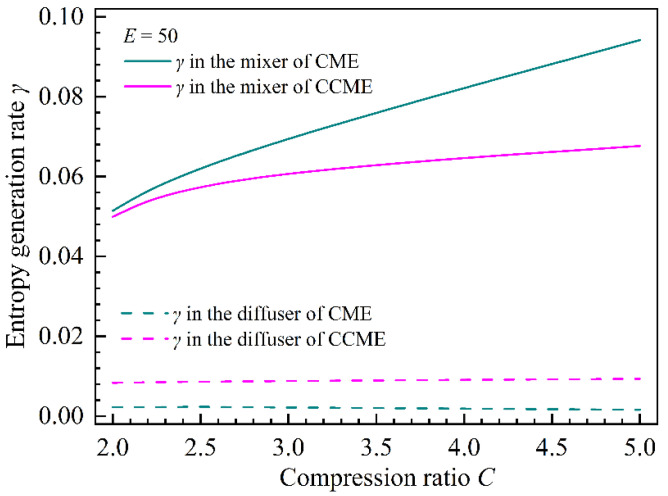
The entropy generation ratio as a function of the compression ratio in the mixer and diffuser of the two types of ejectors.

**Figure 17:**
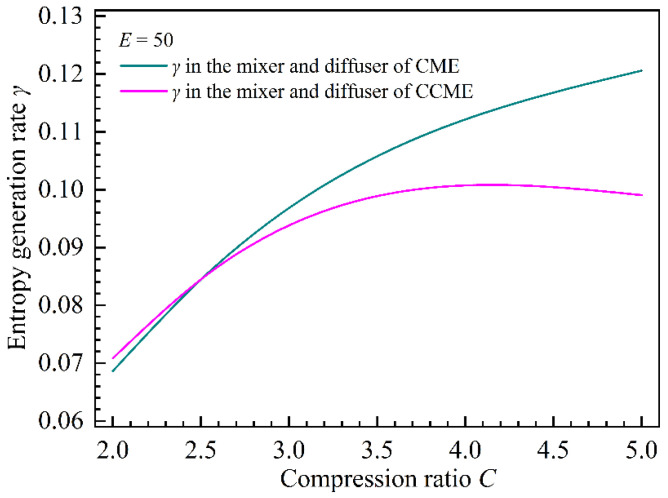
The total entropy generation ratio as a function of the compression ratio in the CME and CCME.

Figure 16 shows the entropy generation rates in the mixer and diffuser of the two types of ejectors under various compression ratios. As the compression ratio increases compared with the CCME, the entropy generation rate in the mixer of the CME increases sharply. The entropy generation rates in the diffusers of the CME and CCME are almost constant, but the entropy generation rates in the diffusers of the CCME are larger. In addition, under small compression ratios, the difference between the entropy generation rate in the CME mixer and that in the CCME mixer is smaller than the difference between the entropy generation rate in the CCME diffuser and that in the CME diffuser. Under large compression ratios, the difference between the entropy generation rate in the CME mixer and that in the CCME mixer is larger than the difference between the entropy generation rate in the CCME diffuser and that in the CME diffuser. This law is more clearly represented in Figure 17 where the distribution characteristics of the entropy generation rates in the mixers and diffusers make the total entropy generation rate in the CME smaller than that in the CCME under small compression ratios, resulting in a better entrainment ratio of the CME. Under large compression ratios, the total entropy generation rate in the CME is larger than that in the CCME, resulting in a better entrainment ratio of the CCME.

#### 4.2.4. Mechanism of the Difference in EGRP in the CCM and CM

From the above analysis, it is evident that the primary distinction in calculating the entrainment ratio between the CME and the CCME resides in the momentum equation of the mixer (refer to Figure 15). Upon comparing Equations (1) and (14), it is observed that the equation for the CCME incorporates an additional term of $\int_{A_{1}}^{A_{d,3}}PdA$, specifically the impulse emanating from the mixer wall. If the magnitude of this term is zero, the mathematical formulations for the mixer in both the CME and CCME become equivalent. The proportion of the impulse from the mixer wall to the overall momentum exiting the mixer (*Mr*) can be mathematically represented as follows:

(36) $$Mr=\frac{\int_{A_{1}}^{A_{d,3}}PdA}{w_{d,3}\left({\dot{m}}_{p}+{\dot{m}}_{s}\right)+P_{d,3}A_{d,3}+\int_{A_{1}}^{A_{d,3}}PdA}$$

**Figure 18:**
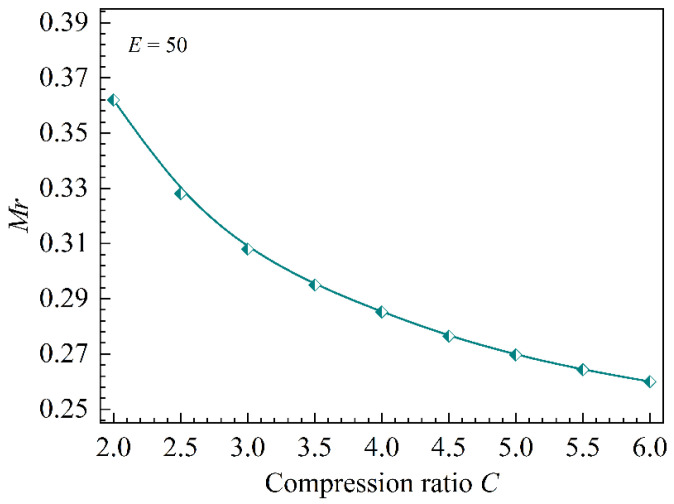
The influence of the compression ratio on *Mr*.

By analyzing the values of *Mr* under a constant expansion ratio (*E* = 50) and varying compression ratios (refer to Figure 18), it is evident that when the CCME is utilized, *Mr* decreases gradually as the compression ratio increases, although at a progressively slower rate. The larger the value of *Mr*, the greater the discrepancy between the momentum equations of the CME and the CCME, which subsequently results in a more significant difference in the EGRP of the mixer.

## 5. Conclusions

To provide a theoretical foundation for selecting between the cylindrical mixer ejector and conical–cylindrical mixer ejector, a comparative study of the evolution laws of the design entrainment ratios in both types of ejectors under various operating conditions was conducted, based on theoretical models validated through experiments. Using the entropy generation rate as the thermodynamic basis for entrainment performance, the influence of mixer structures on the entrainment performance of the ejectors was elucidated. The conclusions drawn are as follows:

1. In the design state, the entrainment ratios of the CME and CCME vary with the compression ratio. The CME exhibits a higher entrainment ratio when the compression ratio is below a certain value, while the CCME performs better when the compression ratio is above this value.
2. After replacing the cylindrical mixer with a conical–cylindrical one, the mixer produces a higher entropy generation rate facing the same pressure lift, while the pressure previously located in the CME’s mixer before replacement transfers to the diffuser, reducing the entropy generation rate from the other side. This leads to an indeterminate efficiency relationship between the pressurization processes of the CME and CCME.
3. As the compression ratio increases, PLPs in the mixers of both the CME and CCME increase slowly, but the EGRPs in the mixer of the CCME decrease more rapidly than those in the CME. The distribution characteristics of PLP and EGRP in the mixer and diffuser result in a smaller total entropy generation rate in the CME compared with the CCME under small compression ratios. Conversely, under large compression ratios, the total entropy generation rate in the CME is larger than that in the CCME.
4. The higher EGRP in the CCME compared with the CME is attributed to the reverse impulse exerted by the contracting wall of the CCME on the incoming mixing flow.

It should be noted that the conclusions of this study are based on the analysis of ejectors with expansion ratios less than 400, which are generally applicable to vapor ejector refrigeration applications. For other applications, such as vacuuming using ejectors, the expansion ratio may exceed 1000. Therefore, further investigation is needed to understand the entrainment ratio advantages and evolution mechanisms of the two types of ejectors under very large expansion ratio conditions.

## Figures and Tables

**Table 1:** The thermodynamic model development for the cylindrical mixer ejector.

Year	Scholars	Conclusions
1942	Keenan et al. [10]	The design model of ejector that satisfies the equations of mass conservation, energy conservation, and momentum conservation is proposed.
1958	Fabri et al. [11]	A formula for calculating flow resistance in circular mixing chamber is introduced.
1965	Paliwoda et al. [12]	The isentropic efficiencies for the nozzle, suction chamber, and diffuser, as well as the momentum loss coefficient for the mixer are introduced.
1989	Sokolov et al. [13]	The optimal working state of the ejector, which occurs at the intersection of the conditions characterized by a double-choking mode and a single-choking mode, is pointed out.
1999	Huang et al. [14]	The correction coefficient for the working flow rate is considered.
2007	Zhu et al. [15]	An exponential two-dimensional velocity distribution model to calculate the entrainment ratio of double-choking mode is introduced.
2012	Cizungu et al. [16]	Calculation models for single-phase and two-phase working fluid nozzles are introduced.
2012	Valle et al. [17]	The Prandtl–Meyer expansion wave is considered to calculate the entrainment ratio and nozzle exit position.
2017	Chen et al. [18]	The theory of expansion wave is used to determine the velocity distribution of the choking section of the secondary vapor.
2018	Kumar et al. [19]	The Fano flow relationship to calculate the axial dimensions of ejectors is introduced.
2020	Tashtoush et al. [20]	The relationship between the momentum loss coefficient in the mixer and the compression ratio, as well as the cross-sectional ratio of the ejector is considered.
2022	Metsue et al. [21]	An ejector model that is based on the properties of real gases and compound-choking theory, which can be applied under both design and off-design operating conditions, is proposed.
2024	Guo et al. [22]	The linear relationship that exists between the mixing loss coefficient and back pressure in the context of single-choking mode is considered.

**Table 2:** The thermodynamic model development for the conical–cylindrical mixer ejector.

Year	Scholars	Conclusions
1950	Keenan et al. [23]	Propose a conical–cylindrical mixer ejector model that is based on momentum, energy, and mass conservation equations.
1977	Munday et al. [24]	Point out that pressurization in the conical–cylindrical mixer is carried out through a shock wave.
1999	Aly et al. [25]	Propose a conical–cylindrical mixer ejector model that combines thermodynamic and aerodynamic methods.
2002	El-Dessouky et al. [26]	Propose using the critical back pressure ratio as the basis for determining whether shock waves occur in the mixer.
2015	Shestopalov et al. [9]	Propose a hypothesis that the secondary vapor choking section coincides with the nozzle outlet section.
2017	Liu et al. [27]	Elaborate on the pressure rise ratio and boosting rate of vapor in the cylindrical sections of the mixer and diffuser.
2023	Wang et al. [28]	Introduce an equivalent equation for the momentum equation of the mixer to reduce the requirement for empirical coefficients in the model.

**Table 3:** The comparative studies between the cylindrical mixer ejector and the conical–cylindrical mixer ejector.

Scholars	Conclusions
Keenan et al. [23]	The CCME may perform better than the CME when the ejector area ratio (*Ar*) is smaller than 10.
Sokolov et al. [13]	The CCME may perform better than the CME when the compression ratio is larger than 2.5.
Shestopalov et al. [29]	Given a generating temperature of *T*_p_ = 88~102 °C and an evaporating temperature of *T*_s_ = 81.6 °C, the CCME with a mixer diameter of 13.02 mm and a nozzle throat diameter of 4.21 mm has a larger entrainment ratio and lower critical back pressure compared with the CME.
Valle et al. [30]	When the mixer diameter is 4.8 mm and the nozzle throat diameter is 4 mm, with *T*_p_ = 84.39 °C and *T*_s_ = 10 °C, the CCME exhibits a larger entrainment ratio and lower critical back pressure compared with the CME.
Shestopalov et al. [9]	Considering the same primary and secondary vapor parameters (*T*_p_ = 95 °C and *T*_s_ = 12 °C), as well as the same condensing temperature of 32 °C, the CCME outperforms the CME when using various organic refrigerants.
Zhu et al. [31]	For an ejector with a primary pressure of *P*_p_ = 5 bar, a secondary pressure of *P*_s_ = 0.43 bar, and a condensing pressure of *P*_d_ = 0.8 bar, the CCME demonstrates a conspicuous performance improvement over the CME.

**Table 4:** Parameters of the pressure sensors.

Parameters	Sensor in Generator	Sensor in Evaporator	Sensor in Condenser
Measuring range	0~350 kPa	0~100 kPa	0~100 kPa
Accuracy	0.075% FS	0.075% FS	0.075% FS

**Table 5:** Uncertainties of the entrainment ratios and compression ratios.

Parameter	Entrainment Ratio	Compression Ratio
Range of uncertainty, %	0.11–0.64	0.72–2.0

**Table 6:** Dimensions of the nozzle.

Nozzle	*D*_p_, mm	*D*_p,0_, mm	*D*_p,1_, mm
NL.1	13.0	2.0	3.9
NL.2	13.0	2.0	6.2
NL.3	13.0	2.0	7.2
NL.4	13.0	2.0	8.2
NL.5	13.0	1.8	8.5
NL.6	13.0	1.8	9.6

**Table 7:** Dimensions of the cylindrical mixing–diffusing chamber.

Mixing–Diffusing Chamber	*D*_suc_, mm	*D*_d,3_, mm	*D*_d_, mm
CM.1	32.0	7.4	15.8
CM.2	32.0	10.2	21.9
CM.3	32.0	12.2	25.6
CM.4	32.0	14.5	30.7
CM.5	32.0	14.5	32.0
CM.6	32.0	15.5	33.4

**Table 8:** Dimensions of the conical–cylindrical mixing–diffusing chamber.

Mixing–Diffusing Chamber	*D*_suc_, mm	*D*_1_, mm	*D*_d,3_, mm
CCM.1	32.0	12.1	7.4
CCM.2	32.0	16.8	10.2
CCM.3	32.0	19.7	12.2
CCM.4	32.0	23.6	14.5
CCM.5	32.0	24.2	14.5
CCM.6	32.0	25.8	15.5

**Table 9:** Comparison of experimental and theoretical entrainment ratios of the CME.

Mixing–Diffusing Chamber	Nozzle	*P*_p_, kPa	*P*_s_, kPa	*P*_d,exp_, kPa	*P*_d,cal_, kPa	Errors	*Er*_exp_	*Er*_cal_	Errors
CM.1	NL.1	19.0	1.23	2.62	2.72	3.82%	0.620	0.614	−0.97%
CM.2	NL.2	70.2	1.23	4.86	4.85	−0.21%	0.270	0.272	0.74%
CM.3	NL.3	101.4	1.23	5.19	5.18	−0.19%	0.275	0.256	−6.91%
CM.4	NL.4	143.4	1.23	5.37	5.39	0.37%	0.289	0.278	−3.81%
CM.5	NL.5	198.7	1.23	6.17	5.95	−3.57%	0.248	0.233	−6.05%
CM.6	NL.6	270.3	1.23	7.36	7.11	−3.40%	0.197	0.173	−12.2%

Note: the primary vapor is 5 °C in superheating, and the secondary vapor is saturated.

**Table 10:** Comparison of experimental and theoretical entrainment ratios of CCME.

Mixing–Diffusing Chamber	Nozzle	*P*_p_, kPa	*P*_s_, kPa	*P*_d,exp_, kPa	*P*_d,cal_, kPa	Errors	*Er*_exp_	*Er*_cal_	Errors
CCM.1	NL.1	19.0	1.23	2.37	2.43	2.53%	0.756	0.764	1.06%
CCM.2	NL.2	70.2	1.23	4.67	4.61	−1.28%	0.363	0.369	1.65%
CCM.3	NL.3	101.4	1.23	4.96	4.86	−2.02%	0.387	0.392	1.29%
CCM.4	NL.4	143.4	1.23	4.91	5.04	2.65%	0.412	0.417	1.21%
CCM.5	NL.5	198.7	1.23	5.60	5.50	−1.79%	0.396	0.411	3.79%
CCM.6	NL.6	270.3	1.23	6.38	6.30	−1.25%	0.363	0.377	3.86%

Note: the primary vapor is 5 °C in superheating, and the secondary vapor is saturated.

## Data Availability

The research data supporting this publication are provided within this paper.

## Nomenclature

*A*	Area, m^2^
*Ar*	Area ratio
*C*	Compression ratio
*D*	Diameter, m
*E*	Expansion ratio
*Er*	Entrainment ratio
*h*	Specific enthalphy, kJ/kg
*k*	Adiabatic index
*L*	Length
$\dot{m}$	Mass flow rate, kg/s
*Mr*	Ratio of the impulse from the mixer wall to the total momentum out of the mixer
*P*	Pressure, Pa
*q*	Flow-rate function of gas dynamic
*s*	Specific entropy, kJ/(kg·K)
*t*	Temperature, K
*v*	Specific volume, m^3^/kg
*w*	Velocity, m/s
**Greek letters**	
*α*	Ratio of the pressure lift in the conical part to the total pressure lift in the conical–cylindrical mixer
*β*	Area ratio of the mixer entrance to the mixer float
*γ*	Entropy generation rate
*η*	Isentropic efficiency
*θ*	Mixer converging angle
*λ*	Speed function of gas dynamic
*μ*	Area ratio of the secondary vapor choking section to the mixer float
*Π*	Pressure function of gas dynamic
*φ*	Coefficient of momentum loss
**Subscripts**	
as	Assumed value
cal	Calculated value
cho	Value in double-chocking mode
cri	Value in critical state
d	Discharge vapor
dif	Parameter in diffuser
exp	Experimental value
id	Ideal value
jet	Value of the jet flow
k	Parameter on cross section k
mix	Parameter in mixing chamber
noz	Parameter in nozzle
p	Primary vapor
s	Secondary vapor
suc	Parameter in suction chamber
0	Parameter in cross section 0
1	Parameter in cross section 1
3	Parameter in cross section 3
**Abbreviations**	
CM	Cylindrical mixer
CCM	Conical–cylindrical mixer
CCME	Conical–cylindrical mixer ejector
CME	Cylindrical mixer ejector
EGRP	Entropy generation rate of per-unit pressure lift
NL	Nozzle
PLP	Pressure lift proportion
FS	Full-scale

## References

[1] National Bureau of Statistics (2021). Energy Statistics Reports for China. https://data.stats.gov.cn/easyquery.htm?cn=C01.

[2] Jing W., Yu J., Luo W., Li C., Liu X. (2021). Energy-saving diagnosis model of central air-conditioning refrigeration system in large shopping mall. Energy Rep..

[3] Liu B., Guo X., Xi X., Sun J., Zhang B., Yang Z. (2023). Thermodynamic analyses of ejector refrigeration cycle with zeotropic mixture. Energy.

[4] Cao X., Liang X., Shao L., Zhang C. (2022). Performance analysis of an ejector-assisted two-stage evaporation single-stage vapor-compression cycle. Appl. Therm. Eng..

[5] Ge J., Chen H., Li J., Jin Y. (2023). Experimental comparison of critical performance for variable geometry ejectors with different mixer structures. Chem. Eng. J..

[6] Eames I.W. (2002). A new prescription for the design of supersonic jet-pumps: The constant rate of momentum change method. Appl. Therm. Eng..

[7] Kitrattana B., Aphornratana S., Thongtip T. (2023). Investigation on improvement potential of steam ejector performance in refrigeration cycle via constant rate of momentum change design method. Appl. Therm. Eng..

[8] Ge J., Chen H., Jin Y., Li J. (2023). Conical-cylindrical mixer ejector design model for predicting optimal nozzle exit position. Energy.

[9] Shestopalov K.O., Huang B.J., Petrenko V.O., Volovyk O.S. (2015). Investigation of an experimental ejector refrigeration machine operating with refrigerant R245fa at design and off-design working conditions. Part 1. Theoretical analysis. Int. J. Refrig..

[10] Kennan J.H., Neumann E.P., Mass C. (1942). A simple air ejector. ASME J. Appl. Mech..

[11] Fabri J., Siestrunck R. (1958). Supersonic air ejectors. Adv. Appl. Mech..

[12] Paliwoda A. (1965). Design problems of supersonic ejectors operating as booster compressors in refrigerating systems. Progress in Refrigeration Science and Technology.

[13] Sokolov E.Y., Zinger N. (1989). Jet Devices.

[14] Huang B., Chang J., Wang C. (1999). A 1-D analysis of ejector performance. Int. J. Refrig..

[15] Zhu Y., Cai W., Wen C., Li Y. (2007). Shock circle model for ejector performance evaluation. Energy Convers. Manag..

[16] Cizungu K., Mani A., Groll M. (2001). Performance comparison of vapor jet refrigeration system with environment friendly working fluids. Appl. Therm. Eng..

[17] Valle J.G., Jabardo J.M.S., Ruiz F.C., Alonso J.S.J. (2012). A one dimensional model for the determination of an ejector entrainment ratio. Int. J. Refrig..

[18] Chen W., Liu M., Chong D., Yan J., Little A.B., Bartosiewicz Y. (2013). A 1D model to predict ejector performance at critical and sub-critical operational regimes. Int. J. Refrig..

[19] Kumar V., Sachdeva G. (2018). 1-D model for finding geometry of a single phase ejector. Energy.

[20] Tashtoush B., Nayfeh Y. (2020). Energy and economic analysis of a variable-geometry ejector in solar cooling systems for residential buildings. J. Energy Storage.

[21] Metsue A., Debroeyer R., Poncet S., Bartosiewicz Y. (2022). An improved thermodynamic model for supersonic real-gas ejectors using the compound-choking theory. Energy.

[22] Guo H., Wang L., Wang X. (2024). A full operating conditions ejector model for refrigeration systems driven by low-grade heat sources. Case Stud. Therm. Eng..

[23] Keenan J.H., Neumann E.P. (1950). An investigation of ejector design by analysis and experiment. J. Appl. Mech..

[24] Munday J.T., Bagster D.F. (1977). A New Ejector Theory Applied to Steam Jet Refrigeration. Ind. Eng. Chem. Process Des. Dev..

[25] Aly N.H., Karameldin A., Shamloul M.M. (1999). Modelling and simulation of steam jet ejectors. Desalination.

[26] El-dessouky H., Ettouney H., Alatiqi I., Al-Nuwaibit G. (2002). Evaluation of steam jet ejectors. Chem. Eng. Process..

[27] Liu J., Wang L., Jia L. (2017). A predictive model for the performance of the ejector in refrigeration system. Energy Convers. Manag..

[28] Wang K., Wang L., Gao R. (2023). An extended mechanism model of gaseous ejectors. Energy.

[29] Shestopalov K.O., Huang B.J., Petrenko V.O., Volovyk O.S. (2015). Investigation of an experimental ejector refrigeration machine operating with refrigerant R245fa at design and off-design working condition. Part 2. Theoretical and experimental results. Int. J. Refrig..

[30] del Valle J.G., Saíz Jabardo J.M., Castro Ruiz F., San José Alonso J.F. (2014). An experimental investigation of a R-134a ejector refrigeration system. Int. J. Refrig..

[31] Zhu Y., Cai W., Wen C., Li Y. (2009). Numerical investigation of geometry parameters for design of high performance ejectors. Appl. Therm. Eng..

[32] Chen H., Lu W., Cao C., Yang L. (2013). Applicability analysis on ejectors with cylindrical and conical mixing chambers. Huagong Xuebao/CIESC J..

[33] Galanis N., Sorin M. (2016). Ejector design and performance prediction. Int. J. Therm. Sci..

[34] Mcgovern R.K., Narayan G.P., John H.L.V. (2012). Analysis of reversible ejectors and definition of an ejector efficiency. Int. J. Therm. Sci..

[35] Luo J., Chen G., Wang Q., Zhang S. (2021). Analysis on the optimal mixing pressure and efficiency limit of an ideal ejector. Energy Rep..

[36] Omidvar A., Ghazikhani M., Razavi S.M.R.M. (2016). Entropy analysis of a solar-driven variable geometry ejector using computational fluid dynamics. Energy Convers. Manag..

[37] Li H., Wang X., Huang H., Ning J., Li A., Tu J. (2022). Numerical study on the effect of superheat on the steam ejector internal flow and entropy generation for MED-TVC desalination system. Desalination.

[38] Liu J., Wang L., Jia L., Wang X. (2018). Thermodynamic modeling and sensitivity analysis of ejector in refrigeration system. Int. J. Heat Mass Tran..

[39] Chen H.J., Zhu J.H., Ge J., Lu W., Zheng L.X. (2020). A cylindrical mixing chamber ejector analysis model to predict the optimal nozzle exit position. Energy.

[40] Sinopec Beijing Design Institute (2000). Specification for Design of Steam-Jet Vacuum Pump for Petrochemical.

[41] Chunnanond K., Aphornratana S. (2004). Ejectors: Applications in refrigeration technology. Renew. Sustain. Energy Rev..

[42] You Y., Deng J.J., Zhang J., Shen Y. (2019). University Physics Experiments.

[43] (1989). General Tolerance. Part 1: Tolerances for Linear and Angular Dimensions without Individual Tolerance Indications.

[44] Nabavi S.R., Wang Z., Rangaiah G.P. (2023). Sensitivity Analysis of Multi-Criteria Decision-Making Methods for Engineering Applications. Ind. Eng. Chem. Res..

[45] Chen H., Zhu J., Lu W. (2018). Optimized selection of one- and two-stage ejectors under design and off-design conditions. Energy Convers. Manag..

[46] Lemmon E.W., Huber M.L., McLinden M.O., Bell I. (2013). Reference Fluid Thermodynamic and Transport Properties Database (REFPROP).

[47] Liu J., Wang L., Jia L., Xue H. (2019). Thermodynamic analysis of the steam ejector for desalination applications. Appl. Therm. Eng..

[48] Khennich M., Sorin M., Galanis N. (2016). Exergy flows inside a one phase ejector for refrigeration systems. Energies.

